# Potential targets and treatments affect oxidative stress in gliomas: An overview of molecular mechanisms

**DOI:** 10.3389/fphar.2022.921070

**Published:** 2022-07-22

**Authors:** Shiyu Liu, Lihua Dong, Weiyan Shi, Zhuangzhuang Zheng, Zijing Liu, Lingbin Meng, Ying Xin, Xin Jiang

**Affiliations:** ^1^ Jilin Provincial Key Laboratory of Radiation Oncology and Therapy, The First Hospital of Jilin University, Changchun, China; ^2^ Department of Radiation Oncology, The First Hospital of Jilin University, Changchun, China; ^3^ NHC Key Laboratory of Radiobiology, School of Public Health, Jilin University, Changchun, China; ^4^ Department of Hematology and Medical Oncology, Moffitt Cancer Center, Tampa, FL, United States; ^5^ Key Laboratory of Pathobiology, Ministry of Education, Jilin University, Changchun, China

**Keywords:** Reactive Oxygen Species (ROS), gliomas, oxidative stress, target gene, therapeutic strategy

## Abstract

Oxidative stress refers to the imbalance between oxidation and antioxidant activity in the body. Oxygen is reduced by electrons as part of normal metabolism leading to the formation of various reactive oxygen species (ROS). ROS are the main cause of oxidative stress and can be assessed through direct detection. Oxidative stress is a double-edged phenomenon in that it has protective mechanisms that help to destroy bacteria and pathogens, however, increased ROS accumulation can lead to host cell apoptosis and damage. Glioma is one of the most common malignant tumors of the central nervous system and is characterized by changes in the redox state. Therapeutic regimens still encounter multiple obstacles and challenges. Glioma occurrence is related to increased free radical levels and decreased antioxidant defense responses. Oxidative stress is particularly important in the pathogenesis of gliomas, indicating that antioxidant therapy may be a means of treating tumors. This review evaluates oxidative stress and its effects on gliomas, describes the potential targets and therapeutic drugs in detail, and clarifies the effects of radiotherapy and chemotherapy on oxidative stress. These data may provide a reference for the development of precise therapeutic regimes of gliomas based on oxidative stress.

## Introduction

Gliomas are common and arise from neuroglial progenitor cells. They are currently incurable central nervous system (CNS) tumors in adults, representing almost 80% of all malignant brain tumors (Ostrom et al., 2014). Glioma incidence and survival rate are associated with numerous factors. Brain tumor development is related to oxidative stress, therefore, it is important to understand oxidative stress mechanisms and develop novel and effective treatments.

In 1990, Sohal et al. first proposed the concept of oxidative stress, either caused by an increase in free radical production or a reduction in the scavenging capacity of the body, leading to disorders in the oxidation and antioxidant systems, resulting in oxidative damage by free radical accumulation (Sohal and Allen, 1990). This process is associated with electron transfer, which affects the redox state of the organism.

The species to which oxygen converts with high reactivity are generally called reactive oxygen species (ROS), which are a type of single electron reduction product of oxygen *in vivo* (Nosaka and Nosaka, 2017). ROS are toxic but are also necessary for regulating the diverse physiological functions of living organisms.

Antioxidative therapy is an effective strategy for many diseases triggered by excess ROS. Low and well-regulated ROS levels enable the functioning of a diverse array of signaling pathways. High levels of ROS-damaged proteins, lipids, and deoxyribonucleic acid (DNA) promote clonal expansion and tumor growth by protecting initial cells from oxidative toxicity and apoptosis (Reczek and Chandel, 2017). Therefore, antioxidative therapy could be used as a research target for glioma treatment. This review describes the existing evidence for the involvement of oxidative stress in the incidence of gliomas, focuses on understanding the function of ROS, and details how to manipulate ROS in glioma treatment.

## Oxidative stress overview

**FIGURE 1:**
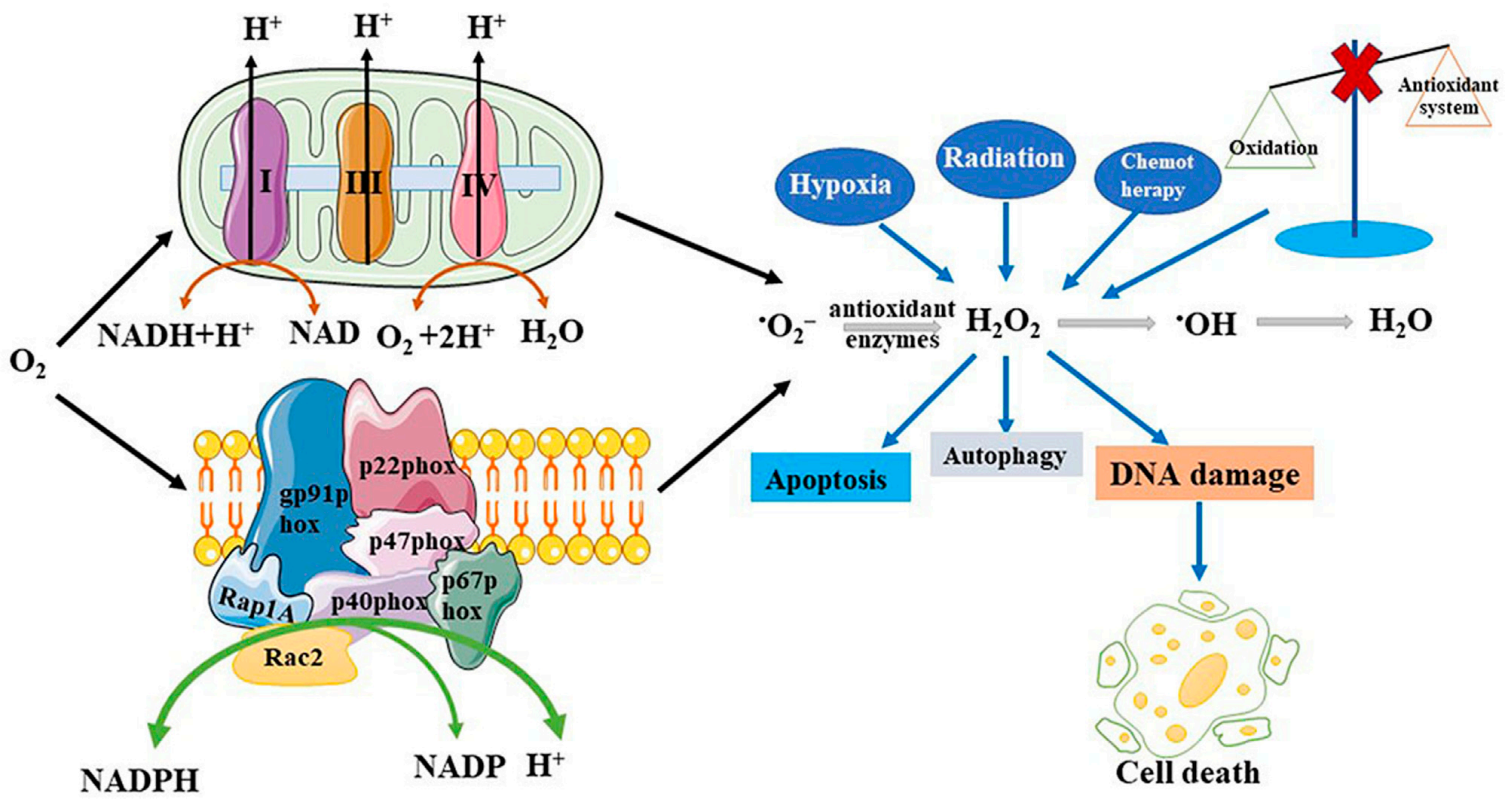
Production and regulation of reactive oxygen species (ROS). ROS can be generated both by the endogenous and exogenous pathways. Endogenous pathway includes the mitochondrial respiratory chain and NADPH oxidase. Exogenous pathway includes hypoxia, chemotherapy, and radiation, among others. When oxidation is imbalanced with antioxidant systems will overproduce ROS. And these pathways lead to disease production, promote DNA damage, increase cell autophagy and apoptosis.

Any atom or molecule containing one or more unpaired electrons is defined as a free radical. ROS is a collective concept consisting of oxygen-based free radicals and some non-radical derivatives of O_2_, including hydrogen peroxide (H_2_O_2_), superoxide anion radicals (^•^O_2_^−^), hydroxyl radicals (•OH), and singlet oxygen (^1^O_2_) (Nosaka and Nosaka, 2017). The regulation of ROS production is shown in Figure 1. ROS have beneficial biological activities and are maintained at appropriate levels by endogenous antioxidant defenses, comprising non-enzymatic antioxidants and antioxidant enzymes. Non-enzymatic antioxidants include tocopherols, ascorbic acid, and glutathione (GSH). Generally, oxidative stress levels are measured using GSH. The antioxidant enzymes include catalase (CAT), superoxide dismutase (SOD), and glutathione peroxidase. Some endogenous pathways can generate ROS, such as the reduction of oxygen molecules during aerobic respiration, resulting in hydroxyl radicals and superoxide. Similarly, the oxidation of catecholamines and the activation of electrons in arachidonic acid co-products reduces oxygen molecules to superoxide (Betteridge, 2000). Many factors stimulate ROS production in various cell types, including cytokines, such as interleukin-1 (IL-1), tumor necrosis factor-α (TNF-α), transforming growth factor-β1 (TGF-β1), interferon-γ (IFN-γ), G protein-coupled receptor binding ligands angiotensin II, serotonin (5-hydroxytryptamine), bradykinin, thrombin, endothelin, and ion channel-linked receptors with neurotransmitters (e.g., acetylcholine, glutamate, glycine, and γ-aminobutyric acid) (Thannickal and Fanburg, 2000). Stimulated by growth factors, ROS act as secondary messenger molecules and initiate a signal cascade in receptor transduction, acting downstream of small guanosine triphosphate (GTP)-binding proteins and receptor tyrosine kinases (RTKs) and upstream of the mitogen-activated protein kinase (MAPK) family (Behrend et al., 2003). The MAPK family mainly consists of c-Jun N-terminal kinases (JNKs: JNK1, JNK2, and JNK3), extracellular signal-regulated kinases (ERKs: ERK1 and ERK2), and p38-MAPKs (p38-MAPKα, p38-MAPKβ, p38-MAPKγ, and p38-MAPKδ) (Wada and Penninger, 2004). JNKs are activated by the phosphorylation of threonine and tyrosine residues catalyzed by MAPK kinase 4 (MKK4) and MKK7, which in turn activate ETS-like protein 1, transcription factor 2, p53, and c-Myc to promote cancer cell proliferation (Wada and Penninger, 2004). Activator protein 1 (AP-1), which is composed of c-Jun and c-Fos, is a downstream transcription factor that is activated by MAPK. It also regulates cyclin D1 and p21 to promote cell proliferation (Waris and Ahsan, 2006). ROS can also be produced through a series of exogenous processes. Exposure to exogenous substances can induce oxidative stress and damage. In the case of ionizing radiation, water decomposes to produce hydroxyl radicals. A study has suggested that the majority of the subversive effects of O_2_ are due to the action of oxygen radicals and an increase in the partial pressure of oxygen or reduction in antioxidant defenses can cause cellular and tissue damage. ^•^O_2_^−^, a free radical, is produced by the monovalent reduction of O_2_ (Gerschman et al., 1954). From a biological perspective, ^•^O_2_^−^ can be generated from two major sources: phagocytic nicotinamide adenine dinucleotide phosphate (NADPH) oxidase and the mitochondrial respiratory chain. Duve and Baudhuin described the process whereby peroxisomes can oxidize the substrate RH2, reducing oxygen to hydrogen peroxide. A large quantity of CAT can reduce hydrogen peroxide to water (O_2_ + RH_2_→R_2_ + H_2_O_2_; H_2_O_2_ + RH_2_→2H_2_O + O_2_ + R) (De Duve and Baudhuin, 1966). Peroxisomes not only participate in ROS generation but also scavenge ROS. Previous studies have shown that NADPH oxidase (NOX) is the principal source of ROS (Brown and Griendling, 2009). NOX is mainly composed of five subunits, including gp91phox (or its homologs, NOX1 and NOX4), p22phox, p47phox, p40phox, and p67phox and two GTP-binding proteins Rap1A and Rac2 (Burtenshaw et al., 2019).

The first SOD that catalyzes the dismutation of superoxide radicals and defends against oxygen free radicals was reported in 1969 (McCord and Fridovich, 1969). Studies have shown that SOD advances the reaction between itself and superoxide anions to form H_2_O_2_ and O_2_ (^•^O_2_^−^ + ^•^O_2_^−^ + 2H^+^→O_2_ + H_2_O_2_) (Fridovich, 1997). H_2_O_2_ reacts with iron ions to generate •OH in Fenton systems, inducing the production of 5,6-dihydroxycytosine, 2,6-diamino-4-hydroxy-5-formamidopyrimidine, 8-hydroxyguanine (8-OHdG) and 4,6-diamino-5-formamidopyrimidine (Halliwell and Gutteridge, 1992). The content of these products can be measured as an index of DNA damage caused by •OH. CAT and peroxidase are inactivated by ^•^O_2_^−^, and SOD reduces the H_2_O_2_ burden borne by aerobic cells by maintaining the activities of peroxidases and CAT (Flint et al., 1993). In any environment where oxygen is produced, the activities of CAT and peroxidase are compromised, and SOD minimizes this effect. However, when ^•^O_2_^−^ is used as an oxidant, it promotes the generation of H_2_O_2_, while SOD prevents chain reactions initiated by ^•^O_2_^−^ oxidation and reduces the generation of H_2_O_2_ (Liochev and Fridovich, 1994).

ROS are well established as playing dual roles as harmful and beneficial components. Overproduction of ROS can induce cell death *via* signaling pathways such as autophagy and apoptosis, resulting in oxidative stress. However, ROS at low or moderate concentrations will exert beneficial effects involving multiple cellular signaling pathways and playing various physiological roles (Valko et al., 2007). Inflammation is a defensive immune response to stimuli, where phagocytes and endothelial cells play a central role and contain ROS generated by NADPH oxidase. Neutrophils also produce ROS that can promote inflammatory cell migration to clear foreign materials and pathogens but this also results in host tissue damage (Mittal et al., 2014). Xanthine oxidoreductase is transformed by proteases into xanthine oxidase, which is then able to transfer electrons from xanthine to oxygen to generate ROS and participate in the inflammatory pathway by inactivating MAPK phosphatase-1, leading to JNK phosphorylation in macrophages (Nomura et al., 2013). Parthanatos, also known as poly ADP-ribose polymerase-1 (PARP-1)-dependent cell death, is a newly described form of programmed brain cell death. JNK phosphorylation promotes oxidative stress-induced parthanatos by increasing intracellular ROS generation (Zheng et al., 2017).

Oxidative stress is associated with several human diseases, including diabetes, cancer, neurodegenerative diseases, cardiovascular diseases, and aging (Aruoma et al., 2006; Milkovic et al., 2014). Increased ROS production leads to disturbances in the balance between oxidation and the antioxidant defense system of the body, causing oxidative stress and this has been observed in cancer cells (Trachootham et al., 2009). Cancer cells exhibit high levels of ROS owing to aberrant signaling, which may be an obstacle to tumor generation. However, ROS can also accelerate tumor growth *via* oncogenic signaling pathways, DNA mutations, and DNA damage (Ames, 1983; Gorrini et al., 2013). DNA mutations are crucial for tumor formation. With the accumulation of ROS, the number of cellular mutations increases, and DNA is constantly damaged. The product of the direct reaction of •OH with guanosine is 8-oxo-7,8-dihydro-2′-deoxyguanosine (8-oxo-dG), which is moderately mutagenic and affects G→T transversion mutations (Fleming and Burrows, 2017). 8-oxo-dG can be an indicator of cellular oxidative stress, with its presence suggesting increased oxidative stress and tumor malignancy. Therefore, the modulation of ROS levels plays a significant role in potential anticancer strategies. Most cancer cells exhibit multiple genetic alterations, increased ROS generation, and altered redox status with additive oxidative stress and aerobic glycolysis, suggesting that preferential clearance of these cells by modulation of the redox modulation mechanism may be a valid strategy for cancer therapy (Trachootham et al., 2009).

## Pathogenesis of gliomas

Gliomas are among the most common malignant brain tumors in adults, accounting for more than 70% of which glioblastoma (GBM) is the most malignant form. GBM accounts for 14.3% of all tumors and 49.1% of malignant tumors (Ostrom et al., 2021). The updated 2016 edition of the World Health Organization (WHO) classification of CNS tumors was the first to use molecular type and histology to define major tumor categories (Louis et al., 2016). This classification divides gliomas into four grades. Grade I mainly includes angiocentric gliomas and pilocytic astrocytomas. Grade II includes diffuse astrocytomas, oligoastrocytomas, and oligodendrogliomas. Grade III includes anaplastic astrocytomas. Finally, grade IV tumors include GBM and gliosarcomas. Traditionally, low-grade gliomas (LGGs) include grade I and grade II gliomas and high-grade gliomas (HGGs) include grade III and grade IV gliomas. However, the fifth edition of the CNS classification adopted in 2021 introduced new types and subtypes of gliomas based on molecular biomarkers (Louis et al., 2021). Currently, the standard treatment for newly diagnosed HGGs is surgical resection within a feasible range, followed by adjuvant radiotherapy (60 Gy/2 Gy/30 f) and concurrent oral temozolomide (TMZ) from the first day of radiotherapy to the last day. Sequential chemotherapy with six cycles of adjuvant temozolomide (Tan et al., 2020). The prognosis of patients with gliomas remains poor despite standard radiotherapy and TMZ treatment. Almost all patients with GBM show disease progression after a median progression-free survival of 7–10 months. Besides radiotherapy and chemotherapy, molecular-targeted therapy is widely used, especially in recurrent gliomas, and holds the promise of providing more effective treatment options with minimal toxicity (Omuro and DeAngelis, 2013). Immunotherapy clears tumors *via* antitumor responses by the host immune system, releases antigens, regulates immune pathways, and elicits tumor-specific cytotoxic T-cells, eventually resulting in immunogenic death (Liu et al., 2021). Despite the current advent of multiple immunotherapies, they have not significantly improved the overall survival of patients with glioma, which is associated with a suppressive immune microenvironment in glioma cells. Immunosuppression of gliomas is associated with multiple biological processes, such as aerobic glycolysis, tryptophan metabolism, and arginine metabolism (Chen and Hambardzumyan, 2018). Multiple mechanisms by which glioma cells evade detection and destruction in the immune system include T-cell, NK cell, and myeloid dysfunction; M2 phenotypic conversion in tumor-associated macrophages/microglia; glioma cell cytokine and surface factor cytokine upregulation; and glioma cell microenvironment hypoxia (Grabowski et al., 2021).

Gliomas are complex microcosms that depend on growth regulatory signals sent by the tumor microenvironment and feature angiogenesis and redox state changes. Communication between non-neoplastic and neoplastic cells contributes to the formation, progression, and response to cancer treatments. Receptors on glioma cells bind to ligands secreted by normal brain parenchymal cells, which may promote glioma invasion or create a microenvironment for malignant progression (Hoelzinger et al., 2007). In addition, abnormal activation of the inflammatory response is a characteristic of GBM and inflammation can promote tumor growth and resistance to treatment (Ham et al., 2019). High ROS levels lead to the death of astrocytes through necrosis and apoptosis, affecting the degree of malignancy *via* the nuclear factor kappa enhancer-binding protein (NF-κB) (Waris and Ahsan, 2006). Cancer development is a multi-stage process described in three stages: initiation, promotion, and progression. The initiation stage involves a non-lethal mutation in the DNA. The promotion phase is a reversible process characterized by the initiation of clonal expansion of cells through the induction of cell proliferation or inhibition of programmed cell death (i.e., apoptosis). At this stage, it is necessary to continue the existence of the tumor to promote stimulation. The final stage of carcinogenesis is irreversible and involves genetic instability and damage to chromosome integrity. The accumulation of additional genetic damage, vascularization, invasion, and metastasis leads to the transformation of cells from benign to malignant, which means that benign preneoplastic lesions become neoplastic cancer cells (Valko et al., 2006).

Oxidative stress is particularly important in glioma pathogenesis. The nervous system is vulnerable to oxidative stress because of high oxygen metabolism in the brain (Barciszewska et al., 2019). ROS-induced oxidative stress leads to DNA damage, which affects the proliferation and apoptosis of glioma cells and increases their susceptibility to gliomas. Human MutT homolog protein 1 (hMTH1) is an enzyme that hydrolyzes 8-oxo-dGTP to the corresponding monophosphate and prevents 8-oxo-dG from accumulating in DNA. The level of oxidative stress is higher in HGGs, therefore, the accumulation of 8-oxo-dG and the expression of hMTH1 are more pronounced. Enhancing the defense against this oxidative stress could be used to treat tumors (Iida et al., 2001). A case-control study showed that the influence of antioxidant gene variations, such as SOD3 T58A, SOD2 V16A, NOS1 3′-UTR, and GPX1-46 C/T, was correlated with the risk of glioma development (Zhao et al., 2012). A study investigated the possible pathway by which H_2_O_2_ induced apoptosis in glioma cells and concluded that oxidative stress inhibited glioma cell growth and induced apoptosis *via* a caspase-3-dependent pathway (Liu et al., 2015). Glioma stem-like cells (GSCs) are a class of subpopulations with stem-like characteristics in glioma cells that confer self-renewal capacity and therapeutic resistance (Mittal et al., 2014). ROS is crucial for the study of therapeutic strategies for GSCs. GSCs, like normal stem cells, maintain low ROS levels, which is in contrast to the high ROS levels in cancer cells (Mittal et al., 2014).

Functional annotation analysis of differentially methylated genes in pediatric GBM and adult GBM identified ROS regulation as a vital process in pediatric GBM and ROS-related genes neutrophil cytosolic factor 1 (NCF1) and NOX4 are upregulated and play important roles in chemosensitivity and proliferation (Jha et al., 2014).

## The mechanism of oxidative stress modulators in gliomas

The design of many molecular targets based on oxidative stress is essential for maximizing survival and transforming this treatment into a form of precision medicine. The following section describes several therapeutic targets that influence oxidative stress.

**FIGURE 2:**
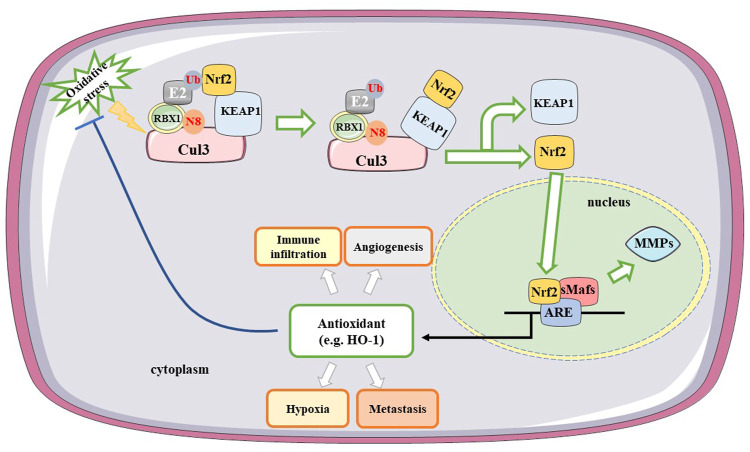
Mechanisms of Nrf2 in treating gliomas. A complex consisting of Cul3, KEAP1, and RBX1 binds to E2 ubiquitin-conjugating enzyme. Under the oxidative stress, Nrf2 induces a conformational change and dissociates from KEAP1, translocating to the nucleus. Nrf2 binds with ARE and heterodimerizes with sMafs. Downregulating Nrf2 expression inactivates MMP-9 and reduces the migration and invasion of gliomas. HO-1 as a downstream factor of Nrf2 participates in immune infiltration, hypoxia, metastasis and angiogenesis. Cul3, Cullin 3; Nrf2, nuclear factor erythroid 2-related factor 2; KEAP1, Kelch-like ECH-associated protein 1; RBX1, ring-box-1; ARE, antioxidant response elements; MMPs, matrix metalloproteinases; H O -1, heme oxygenase 1; sMafs, small musculoaponeurotic fibrosarcoma proteins; N8, Nedd8; Ub, Ubiquitination.

Oxidative stress activates multiple transcription factors, including hypoxia-inducible factor-1α (HIF-1α), AP-1, NF-κB, p53, and nuclear factor erythroid 2-related factor 2 (Nrf2) (Reuter et al., 2010). Nrf2 is an important component of the cellular defense against various exogenous and endogenous stresses that can be activated in response to a series of oxidative and electrophilic stimulations (Kensler et al., 2007). Nrf2 serves as a potential therapeutic target in gliomas since activating its expression will increase the content of target antioxidants and enzymes that protect cells from apoptosis, whereas inhibiting its expression can elevate the killing effects of antitumor therapies (Zhu et al., 2014). Kelch-like ECH-associated protein 1 (KEAP1) is an inhibitor of Nrf2, which acts by modulating Nrf2 activity. A complex consisting of Cullin 3 (Cul3), KEAP1, and ring-box 1 (RBX1) binds to E2 ubiquitin-conjugating enzyme, and Nrf2 and Nedd8 (N8) induce a conformational change that inhibits Nrf2 ubiquitination (Baird and Yamamoto, 2020). Upon recognition of oxidative stress, Nrf2 dissociates from KEAP1, translocates to the nucleus, and heterodimerizes with small musculoaponeurotic fibrosarcoma proteins (sMafs). Nrf2 and other transcription factors regulate the expression of antioxidant genes by interacting with antioxidant response elements (ARE) (Reuter et al., 2010). The KEAP1–Nrf2–ARE signaling pathway plays a significant role in protecting cells from oxidative stress. Oxidative stress-related molecules and matrix metalloproteinases (MMPs) are involved in regulating glioma migration and invasion *via* the Nrf2/ARE pathway (Deryugina et al., 1997). Downregulation of Nrf2 expression can inactivate MMP-9 and reduce the migration and invasion of gliomas (Pa n et al., 2013). Heme oxygenase-1 (HO-1), a downstream molecule of Nrf2, plays a key role in regulating oxidative stress. Nuclear Nrf2 upregulates HO-1 and decreases intracellular ROS (Kanzaki et al., 2013). HO-1, which is involved in heme metabolism, catalyzes the conversion of heme to biliverdin and generates carbon monoxide during this process (Hayashi et al., 1999). HO-1 protein expression is associated with the degree of glioma malignancy and is overexpressed in HGGs. Moreover, HO-1 participates in immune cell infiltration and is associated with metastasis and angiogenesis. The mechanism of action of Nrf2 in glioma treatment is shown in Figure 2.

HIF-1 is a DNA-binding protein and is composed of two different subunits, 120 kDa HIF-1α and 91–94 kDa HIF-1β (Wang and Semenza, 1995). HIF-1 allows tumor cells to survive in the absence of oxygen, activating the transcription of glycolytic enzymes, glucose transporters, and vascular endothelial growth factors (Zhong et al., 1999). ROS can alter the function and activity of HIF-1 and inhibition of HIF-1 activity contributes to tumor therapy. NOX4 mRNA expression levels in GBM are markedly higher than those in other astrocytomas (Shono et al., 2008). A previous study revealed that circulating hypoxic conditions increase ROS production, activate HIF-1, and promote the growth of glioma cells by upregulating the expression of NOX4 mRNA and protein expression in GBM cells (Hsieh et al., 2011). NOX2, a downstream target gene of microRNA (miR)-34a, increases ROS levels and promotes apoptosis in glioma cells (Li et al., 2014). Therefore, knockdown of NOX2 and NOX4 during GBM progression may be a therapeutic method for counteracting the effect of hypoxia on tumor progression.

A study identified that diacylglycerol O-acyltransferase 1 (DGAT1) is highly expressed in HGGs. Inhibition of DGAT1 was shown to significantly upregulate the carnitine palmitoyltransferase 1A (CPT1A) protein, which facilitates the entry of excessive fatty acids (FAs) into the mitochondria for oxidation, resulting in mitochondrial damage, remarkable increase in GBM cell apoptosis, and ROS production (Cheng et al., 2020). Therefore, targeting DGAT1 may be a potential therapeutic approach for glioma treatment.

**FIGURE 3:**
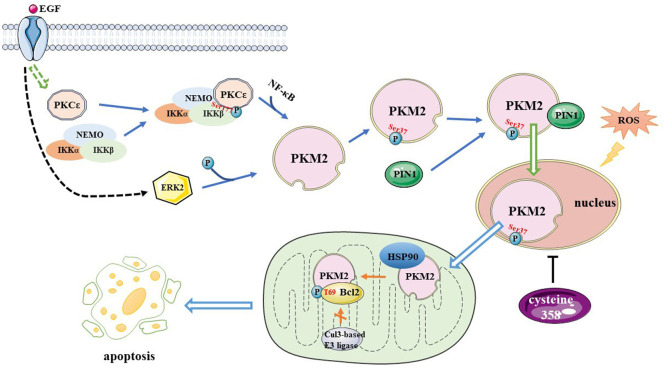
A mechanism for PKM2 regulation. EGF induces PKCε generated to phosphorylate IKKβ at Ser177, increasing PKM2 expression, and NF-κB is involved in this process. EGF also activates ERK2, which phosphorylates PKM2 at the Ser37 site, allowing PIN1 to bind to it, it also promotes PKM2 translate to nucleus. Oxidative stress induces PKM2 translocation to mitochondria and phosphorylates Bcl2 at T69 site with the help of Hsp90, preventing the Cul3-based E3 ligase from binding to Bcl2 and increasing the resistance of glioma cells to oxidative stress-induced apoptosis. Cysteine 358 oxidation inhibits PKM2 activity, activating the reactive oxygen species scavenging system. PKM2, pyruvate kinase M2; EGF, Epidermal growth factor; PKC, protein kinase C; IKK, IκB kinase; NF-κB, nuclear factor kappa enhancer binding protein; ERK, extracellular signal-regulated kinase; Bcl2, B-cell lymphoma 2; T, threonine; HSP90, Heat Shock Protein 90; Cul3, cullin 3

Epidermal growth factor receptor (EGFR) induces protein kinase Cε (PKCε) to phosphorylate and activate IκB kinase β (IKKβ) at Ser177, increasing the expression of pyruvate kinase M2 (PKM2). NF-κB is also involved in this process (Yang et al., 2012a). EGFR also induces ERK2 to phosphorylate PKM2 at Ser 37, which allows peptidylprolyl cis/trans isomerase NIMA-interacting 1 (PIN1) to bind to PKM2, prompting PKM2 to translocate to the nucleus, upregulating lactate dehydrogenase A (LDHA) and glucose transporter 1 (GLUT1) expression and promoting the Warburg effect (Yang et al., 2012b). A previous study indicated that the expression of PKM2 is correlated with the grade of glioma malignancy and that the level of PKM2 is lower in LGGs than in HGGs (Yang et al., 2012a). The heat shock protein (HSP) 90–PKM2–B-cell lymphoma 2 (Bcl2) axis is a potential therapeutic target in GBM treatment. In cancer cells, PKM2 affects ROS levels in two ways (Liang et al., 2017). Firstly, oxidative stress induces PKM2 translocation to the mitochondria where it phosphorylates Bcl2 at Thr69 site with the help of the chaperone protein HSP90α1. This prevents the combination of Cul3-based E3 ligase and Bcl2, thereby maintaining the stability of Bcl2 and increasing the resistance of glioma cells to oxidative stress-induced apoptosis. Researchers have also found that the PKM2 389–405 peptide is an efficacious medicine that disrupts the interaction between PKM2-Bcl2 leading to an antitumor effect that hinders the development of gliomas. Secondly, Cys358 oxidation inhibits PKM2 activity, thereby activating the ROS scavenging system in response to increased ROS levels. Collectively these results indicate that PKM2 could be a potential target for developing effective treatment of GBM. The mechanism underlying PKM2 regulation is shown in Figure 3.

Protein tyrosine phosphatase non-receptor type 2 (PTPN2) was recently identified as a novel cancer target. PTPN2 is oxidized and inactivated by H_2_O_2_ and the expression levels of PTPN2 are increased in GBM and isocitrate dehydrogenase (IDH) wild-type gliomas. An increase in PTPN2 levels is correlated with a worse overall survival rate (Wang et al., 2018). Furthermore, another study observed this phenomenon, indicating that oxidative stress may be exploited to stimulate PTPN2 inactivation for treating gliomas (Wu et al., 2019).

Prohibitin (PHB) is a highly conserved pleiotropic protein that plays a vital role in multiple biological processes. Peroxiredoxin3 (PRDX3) is a specific peroxidase in the mitochondria that scavenge peroxides and protects cells from oxidative stress. PHB binds to and stabilizes PRDX3 to inhibit mitochondrial ROS accumulation and promote GSCs self-renewal. Therefore, knockout of the PHB gene significantly increases ROS levels and inhibits GSCs self-renewal (Huang et al., 2021).

The oncostatin M receptor (OSMR) is a direct signal transducer and activator of the transcription 3 (STAT3) target gene, a member of the IL-6 receptor family, and is involved in many cellular responses, such as differentiation, proliferation, and survival. The depletion of OSMR affects EGFRvIII–STAT3 signaling and significantly retards the proliferation of GBM cells, prolonging their lifespan (Jahani-Asl et al., 2016). A study examined the relationship between ROS and OSMR and found interaction with nicotinamide adenine dinucleotide (NADH) ubiquinone oxidoreductase 1/2 (NDUFS1/2). Deleting OSMR promotes the generation of ROS, sensitizes GBM cells to radiotherapy, and induces glioma cell death (Sharanek et al., 2020). It is possible to identify drugs that inhibit OSMR expression to achieve the goal of treating gliomas.

Paired box 6 (PAX6) is a DNA-binding transcription factor that downregulates the expression of the vascular endothelial growth factor A (VEGFA) gene in glioma cells to suppress tumor cell invasion. PAX6 expression was found to be significantly reduced in GBM compared to LGGs. It has been shown that GBM cells with lower PAX6 levels survive better in a stressful environment after detachment from the culture. ROS levels increased following cell detachment and the addition of antioxidants enhanced the viability of PAX6-overexpressing cells, however, this did not recover their proliferative capacity (Chang et al., 2007).

A study that utilized proteomic analysis of cells from patients with GBM revealed that the autocrine factor midkine (MDK) promotes cell proliferation and detoxifies ROS. Inhibition of MDK expression may serve as a novel approach for GBM treatment by inducing ROS-mediated apoptosis and cell cycle arrest (Han et al., 2019).

Proteomics suggests that HOXA transcript antisense RNA myeloid-specific 1 (HOTAIRM1) is associated with mitochondrial function, and knockdown of HOTAIRM1 can increase the level of ROS and radiation sensitivity, thereby prolonging patient survival (Ahmadov et al., 2021).

Apurinic/apyrimidinic endonuclease1 (APE1), associated with checkpoint kinase 2 (Chk2), participates in the coordination of double-strand break DNA repair. Ectonucleotide pyrophosphatase/phosphodiesterase 2 (ENPP2) is a secreted protein that possesses lysophospholipase D activity and hydrolyzes pyrophosphate bonds and phosphodiesters from various substrates (Amaral et al., 2021). Oxidative stress elevates the expression levels of APE1 and PKM2 and stimulates the extracellular secretion and intracellular expression of ENPP2 in glioma cells (Cholia et al., 2018). These results revealed that glioma progression is mediated by the regulation of activity, expression levels, and the correlation of these three enzymes. Sirtuin 6 (SIRT6) is a nuclear NAD^+^-dependent histone H3 deacetylase that regulates its genomic expression and stability. An experiment revealed that mir-33a reduced ROS levels by inhibiting SIRT6 expression and decreasing cell survival following H_2_O_2_ treatment (Chang et al., 2017). SIRT6 suppresses the oxidative stress response while inhibiting Janus kinase 2 (JAK2)/STAT3 signaling pathway activation during glioma treatment (Feng et al., 2016). Recent studies on the target genes that influence oxidative stress in gliomas are summarized in Table 1.

**TABLE 1:** Summary of studies on target genes linked to ROS in gliomas.

Target gene	Expression in gliomas	Regulatory pathway	Result	Survival	Study design	References
*DGAT1*	High	DGAT1/CPT1A/FAs	ROS↓ apoptosis↓	Low	*in vivo, in vitro*	Cheng et al. (2020)
*PKM2*	High	HSP90/PKM2/Bcl2	oxidative stress induced apoptosis↓	Low	*in vivo, in vitro*	Liang et al. (2017)
*PTPN2*	High	STAT/PTPN2	PTPN2 was inactivated and oxidated by ROS	Low	*in vitro*	Wu et al. (2019)
*OSMR*	High	OSMR/NDUFS1/2	mitochondrial respiration↑ ROS↓	Low	*in vivo, in vitro*	Sharanek et al. (2020)
*SIRT6*	Low	SIRT6/JAK2/STAT3	cell injury↑ ROS↓ cell growth↓	High	*in vitro*	Feng et al. (2016)
*SIRT6*	Low	miR-33a/SIRT6	apoptosis↑	High	*in vitro*	Chang et al. (2017)
*PHB*	High	miR-27a/PHB/peroxiredoxin3 (PRDX3)	ROS↓ cell growth↓ Radioresistance↓	Low	*in vivo, in vitro*	Huang et al. (2021)
*PRDM16*	High	miR-101/DNMT3A/PRDM16/H3K27me3 H3K4me2	ROS↑ apoptosis↑	Low	*in vitro*	Lei et al. (2016)
*HERPUD1*	High	miR-9-3p/Herpud1	H_2_O_2_ induced apoptosis↓	Low	*in vitro*	Yang et al. (2017)
*ATF4*	High	ATF4/xCT/SCL7A11	tumor cell growth↑ xCT transporter activity↑ ferroptosis↓ ROS↓	Low	*in vitro*	Chen et al. (2017)

## The efficacy of phytocompounds in gliomas

Several studies have emphasized the relationship between oxidative stress and the emergence of drugs. However, many drugs are unable to cross the blood–brain barrier (BBB) to achieve maximum therapeutic efficacy. The BBB is composed of capillary endothelial cells, an intact basement membrane, and glial membranes surrounding astrocyte foot plates and is a barrier between the walls of brain capillaries and plasma to brain cells formed by glial cells. The BBB excludes substances that are hazardous to the brain, protects the brain from harm, and allows particles smaller than 20 nm in diameter to cross over (Abbott et al., 2010). If a drug is converted into a small molecule, it can pass through the BBB to achieve the purpose of treatment.

Quinoxaline-1,4-dioxide derivatives are a class of synthetic heterocyclic compounds that exhibit diverse biological and pharmacological effects. They can promote cell damage by increasing ROS (Silva et al., 2019). Thymoquinone (TQ) is a drug that can penetrate the BBB and act against gliomas through its antioxidant, antimetastatic, and anti-invasive activities (Racoma et al., 2013). TQ regulates the production of superoxide in mitochondria in a dose-dependent manner and low-dose TQ inhibits superoxide production in mitochondria. ROS generation has been shown to increase with higher TQ concentrations. It has also been confirmed that TQ induces apoptosis in C6 glioma cells *via* redox-dependent MAPK proteins (Krylova et al., 2019). This provides direction for treatment options for gliomas.

Chidamide is a histone deacetylase (HDAC) inhibitor that selectively inhibits the activity of HDAC1, 2, 3, and 10 (Shi et al., 2017). The Hedgehog (Hh) signaling pathway affects glioma growth. This pathway is initiated by a combination of Patched and Hh proteins, consisting of Sonic Hh (Shh), Desert Hh, and Indian Hh (Ihh), which allows Smoothened to transmit signals to the nucleus. A basic study suggested that chidamide inactivates Hh signaling by increasing the level of miR-338-5p, increasing oxidative stress and promoting glioma cell apoptosis and necrosis (Zhou et al., 2020). Chidamide could therefore be used as a potential drug to prevent glioma development.

A review summarized that combining antiparasitic drugs, such as ivermectin, atovaquone, proguanil, quinacrine, and mefloquine with radiotherapy could potentially enhance the radiosensitivity of HGGs by abolishing tumor hypoxia and enhancing oxidative stress (Mudassar et al., 2020). In conclusion, the combination of radiotherapy and antiparasitic drugs may be a new method for the treatment of malignant HGGs and may improve patient survival.

Quercetin (Qu), a plant-derived flavonoid, is known for its anti-tumor and anti-proliferative activities. Qu has been modified as a chemoprotective and radiosensitive agent that plays an important role in the treatment of GBM, and it has been found to inhibit oxidative stress by scavenging ROS to achieve antitumor effects (Tavana et al., 2020).

One study examined the antitumor effects of eight Cu(II) complexes with uracil-functionalized ligands in glioma cells. These compounds promoted apoptosis and autophagy in glioma cells by affecting the activities of SOD and CAT (Illán-Cabeza et al., 2020). Thus, copper (II) complexes can be used as drugs to manipulate the redox microenvironment to treat gliomas.

Melatonin, a free-radical scavenger, exerts antioxidant effects and protects the BBB under hypoxic conditions. Melatonin has been shown to significantly reduce the invasion and migration of glioma cells by inhibiting the ROS/NF-κB/MMPs pathway (Wang et al., 2012). This indicates that melatonin has potential therapeutic applications for the treatment of gliomas.

TNF-related apoptosis-inducing ligand (TRAIL), a member of the TNF superfamily, induces apoptosis by binding to receptors that contain death domains. The fungal metabolite chaetocin is a novel TRAIL sensitizer and is an inhibitor of the histone methyltransferase SUV39H1. Chaetocin promotes apoptosis by depleting the expression of heme oxygenase 1 (HMOX1) thereby inducing ROS-dependent apoptosis (Ozy erli-Goknar et al., 2019). The increase in ROS can also affect the apoptosis and metabolism of glioma cells *via* the JNK-regulated metabolic pathway and ataxia telangiectasia mutated (ATM)-Yes-associated protein 1 (YAP1)-driven apoptotic pathway (Dixit et al., 2014). These results provide a basis for the combination of chaetocin and TRAIL for the treatment of gliomas.

Celastrol is one of the most important active ingredients of the traditional Chinese medicine *Tripterygium wilfordii*, which activates the JNK pathway and ROS production and inhibits the activities of mechanistic targets of rapamycin (mTOR) and Akt kinases, significantly increasing apoptosis and autophagy in glioma cells (Liu et al., 2019).

Osthole is a coumarin derived from traditional Chinese medicine. One study found that osthole is a potential drug for treating gliomas, as it increases the production of ROS and upregulates the expression of induced receptor interacting protein kinase 1 (RIP1), RIP2, and mixed lineage kinase domain-like protein (MLKL). These results confirm that osthole induces mitochondrial depolarization and necroptosis (Huangfu et al., 2021).

Shikonin, a naphthoquinone, has been studied as a preventive or therapeutic drug for the treatment of gliomas. Shikonin dose-dependently induces ROS overproduction in glioma cells and upregulates RIP1 and RIP3 to mediate necroptosis (Lu et al., 2017).

Small molecule antioxidants containing selenium can ameliorate oxidative damage. Selenocysteine (SeC), a naturally available selenoamino acid that potentiates the production of ROS and superoxide anions, induces DNA damage, causes S-phase cell cycle arrest, and inhibits the growth of glioma cells (Wang et al., 2016).

An experiment revealed that polyphyllin VI (PPVI), a bioactive ingredient extracted from the traditional Chinese medicine *Paris polyphylla*, increases ROS accumulation, which in turn activates ROS-regulated JNK and p38 pathways and regulates the G2/M phase to inhibit glioma cell proliferation (Liu et al., 2020). Thus, PPVI might be a potential therapeutic agent for gliomas.

Deoxypodophyllotoxin (DPT), isolated from herbal plants, is used as a precursor for teniposide and etoposide phosphate. Overproduction of DPT triggers ROS-induced upregulation of PARP-1, which promotes apoptosis-inducing factor (AIF) translocation into the nucleus, causing parthanatos in glioma cells (Ma et al., 2016). This study provides novel insights for the development of an anti-glioma strategy.

Cannabidiol (CBD) is a non-psychoactive, natural ingredient extracted from cannabis. CBD has proapoptotic and antiproliferative effects and serves an anti-glioma purpose by increasing the production of ROS and the activity of GSH-associated enzymes, as well as depleting glutathione (Massi et al., 2006). Another study found a similar view that CBD induces a substantial increase in ROS, thereby inhibiting GSCs survival and self-renewal (Singer et al., 2015).

Proteomic analysis of cells following treatment with loperamide and pimozide revealed that these drugs can induce endoplasmic reticulum stress, leading to increased ROS levels and promoting cell death (Meyer et al., 2021).

**FIGURE 4:**
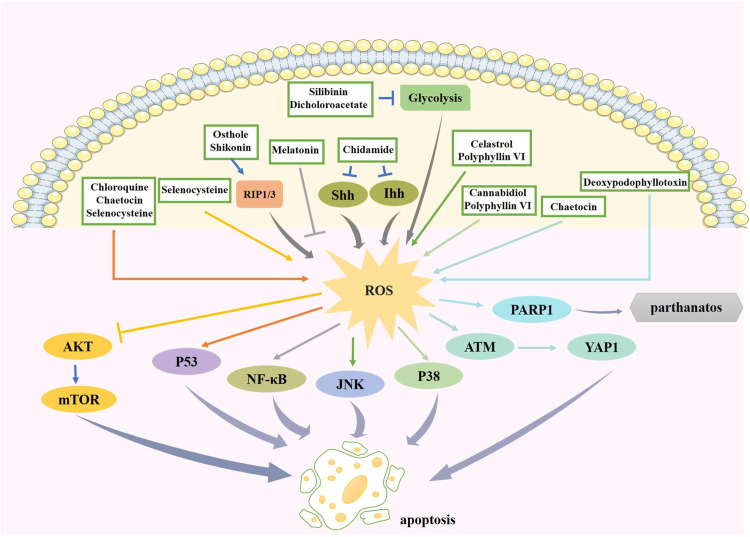
The effect and pathway of available therapeutic agents in glioma pertinent to ROS. Chidamide increases ROS production through the Hh signaling pathway. Melation promotes apoptosis by suppressing ROS production, leading to the reduction of its downstream factor NF-κB. Chaetocin promotes ROS production and can promote apoptosis through the p53 pathway or the ATM/YAP1 pathway. Celastrol and polyphyllin VI promote ROS generation to induce apoptosis *via* JNK pathway. Osthole and shikonin increase ROS by promoting RIP1/3 expression. Increased ROS expression by selenocysteine inhibited Akt pathway on one hand and activated p53 pathway on the other hand to promote cell apoptosis. Increased ROS expression by polyphyllin VI and cannabidiol activates p38 pathway to induce apoptosis. Deoxypodophyllotoxin increases the expression of ROS and thus PARP1 expression, causing parthanatos. Silibinin and dicholoroacetate can have inhibition of glycolysis and increase ROS expression. ROS, reactive oxygen species; NF-κB, nuclear factor kappa enhancer binding protein; JNK, c-Jun N-terminal kinase; RIP1/3, receptor interacting protein kinase 1/3; ATM, ataxia telangiectasia mutated; YAP1, Yes-associated protein 1; PARP-1, poly ADP-ribose polymerase-1.

Silibinin is a polyphenolic extract of *Silybum marianum*. Silibinin suppresses glycolysis in tumor cells, thereby activating autophagy. Autophagy increases H_2_O_2_ levels by promoting p53-mediated GSH depletion and inducing Bcl2 interacting protein 3 (BNIP3) upregulation, mitochondrial damage, and AIF translocation from the mitochondria to the nucleus, resulting in glioma cell death (Wang et al., 2020). Therapeutic agents that regulate ROS levels to provide new ideas for glioma treatment are summarized in Table 2. And the effect and pathway of these therapeutic agents pertinent to ROS are shown in Figure 4.

**TABLE 2:** Summary of studies on medicines linked to ROS in gliomas.

Medicine	Type	Study design	Cell	Pathway	Result	Reference
Thymoquinone	Chemotherapeutic agent	*in vitro*	C6	PI3K/AKT	Proliferation↓ ROS↑ Apoptosis↑	Krylova et al. (2019)
Chidamide	HDAC inhibitor	*in vitro*	U87; HS683	miR-338-5p/ Hedgehog	ROS↑ Proliferation↓ Migration↓ Invasion↓	Zhou et al. (2020)
Atovaquone	Anti-malarial drug	*in vivo, in vitro*	U87-MG; LN-18, SF-188; SJ-GBM2	STAT3	ROS↑ Apoptosis↑	Takabe et al. (2018)
Ivermectin	Anthelmintic drug	*in vitro*	U87; T98G	Akt/mTOR	Angiogenesis↓ Cell growth↓ ROS↑	Liu et al. (2016)
Chloroquine	Anti-malarial drug	*in vitro*	U87; LN308; U118; U251; LN229	P53	ROS↑ Autophagic vacuoles accumulation	Geng et al. (2010)
Quinacrine	Antiprotozoal agent	*in vivo, in vitro*	C6; GSCs	Ras/MAPK	Survival period↑ ROS↑	Wang et al. (2017)
Quercetin	Flavonoid	*in vitro*	C6	-	oxidative stress↓	Ersoz et al. (2020)
Melatonin	Indolamine	*in vitro*	T98G; U251	NF-κB/MMPs	ROS↓ migration↓ invasion↓	Wang et al. (2012)
Chaetocin	Fungal metabolite	*in vitro, in vivo*	U87MG; U373; T98G	HMOX1/TRAIL;P53	ROS↑ apoptosis↑	Ozy erli-Goknar et al. (2019)
Celastrol	Triterpene compound	*in vitro, in vivo*	U251; U87-MG; C6	ROS/JNK Akt/mTOR	G2/M phase arrest; ROS, apoptosis and autophagy↑	Liu et al. (2019)
Osthole	Coumarin derivative	*in vitro*	U87; C6	RIP1/RIP3/MLKL	ROS↑ necroptosis↑	Huangfu et al. (2021)
Shikonin	Naphthoquinone	*in vitro*	C6; SHG-44; U87; U251	RIP1/RIP3	ROS↑ necroptosis↑	Lu et al. (2017)
Selenocysteine	Selenoamino acid	*in vitro*	U251; U87	MAPK/Akt	ROS↑ DNA damage↑	Wang et al. (2016)
Polyphyllin VI	Component derived from Chinese herb Paris polyphylla	*in vivo, in vitro*	U251; U343; LN229; U87; HEB	JNK/P38	ROS↑ autophagy↑ apoptosis↑ cell cycle arrest	Liu et al. (2020)
Chaetocin	A histone methyltransferase inhibitor	*in vivo, in vitro*	A172; T98G; U87-MG	ATM/YAP1; JNK	ROS↑ apoptosis↑	Dixit et al. (2014)
Deoxypodophyllotoxin	Major lignan of plant Anthriscus sylvestris phosphate	*in vivo, in vitro*	C6; SHG-44; U87	PARP1	ROS↑ cell death↑	Ma et al. (2016)
Cannabidiol	A non-toxic, non-psychoactive cannabinoid	*in vivo, in vitro*	U251; GSC lines 387 and 3832	p-p38	ROS↑ GSC survival↓ self-renewal↓ invasion↓	Singer et al. (2015)
Silibinin	A polyphenolic extract from silybum marianum	*in vivo, in vitro*	U87; U251; SHG-44; C6	Glycolysis; P53	GSH↓; H_2_O_2_↑; BNIP3↑	Wang et al. (2020)
Dicholoroacetate	Glycolytic inhibitor	*in vivo, in vitro*	Gl261; U-87 MG; U-251 MG; T98G	Glucose and FAO metabolic pathways	ROS↑ autophagy↑ DNA damage↑ apoptosis↑	McKelvey et al. (2021)
Ranolazine	Partial fatty acid oxidation inhibitor					
MNPC	NADPH quinone oxidoreductase 1 (NQO1) and glutathione-S-transferase Pi 1 (GSTP1) inhibitor	*in vivo, in vitro*	U87MG/EGFRVIII; U87-MG	PTEN/NQO1 GSTP1/ PI3K/Akt	Oxidative stress↑ apoptosis↑	Lei et al. (2020)

## Effect of radiotherapy and chemotherapy on oxidative stress in gliomas

Radiotherapy and chemotherapy, as standard treatment strategies, have been rapidly developed and are widely used in clinics to eliminate gliomas. However, resistance to radiation and chemotherapeutic drugs is a fundamental obstacle to improving the curative effect of gliomas. Therefore, the design and development of novel chemoradiotherapy strategies to overcome resistance have become a focus of clinical oncology research. Gliomas need to use radiotherapeutic or chemotherapeutic drugs to influence the prognosis through ROS modulation. A study demonstrated that combined treatment with radiation and salinomycin (SAL) increased DNA damage and tumor apoptosis by increasing ROS production, which is a novel strategy to improve the efficacy of radiotherapy in cancer prevention and overcome radioresistance (Liu et al., 2018). The radioresistance of human glioma cells induced by SOD1 overexpression is related to the inhibition of late ROS accumulation and enhancement of G2/M accumulation (Gao et al., 2008). Another study revealed that adenosine triphosphate (ATP) channels can control glioma radioresistance by adjusting ROS-induced ERK activation; thus, inhibiting ATP channels is a potential target for glioma therapeutic development (Huang et al., 2015). One way to increase radiosensitivity is to increase intracellular ROS by 5-aminolevulinic acid treatment, which results in the radiosensitization of glioma cells (Kitagawa et al., 2015). The transcriptional activity of the HIF-1 signal induced by ROS in cyclic hypoxia was higher than that in intermittent hypoxia. Under hypoxic conditions, knockout of the HIF-1 gene inhibits uninterrupted hypoxia-induced radioresistance while increasing the overall radiosensitivity of the tumor (Hsieh et al., 2010). Outer-membrane vesicles (OMVs) from *Escherichia coli* and gold nanoparticles (AuNPs) were combined to synthesize Au-OMVs. Combining Au-OMVs with radiotherapy generated ROS to increase radiosensitization and suppress glioma cell growth (Chen et al., 2021). Proton beam radiation generates substantial amounts of ROS, which induces cell cycle redistribution and DNA damage and promotes apoptosis in GSCs (Alan Mitteer et al., 2015). The main adverse effect is a radiation-induced skin reaction, with its mechanisms including inflammation and oxidative stress, which interact and promote each other. Direct exposure of normal cells to radiation or ROS may lead to apoptosis and necrosis, which triggers the release of anti-inflammatory cytokines (Wei et al., 2019).

During chemotherapy, when O^6^-methylguanine methyltransferase, alkylpurine-DNA-N-glycosylase, and base excision repair proteins are expressed, GBM cells are resistant to TMZ (Lee, 2016). Drug efflux transporters, the advent of GSCs, and the upregulation of autophagy are also mechanisms of TMZ resistance (Tomar et al., 2021). The curcumin analog ALZ003 increased the production of ROS and ubiquitinated the androgen receptor resulting in its degradation, which potentiated TMZ resistance. This result provides evidence to improve the efficacy in glioma patients resistant to TMZ (Chen et al., 2020). Gemcitabine combined with nanomaterials, such as AgNTs, participates in ROS-dependent mitochondrial pathway-mediated apoptosis, thereby inhibiting the activity of gliomas, indicating that AgNTs and chemotherapeutics have a synergistic effect (Yang et al., 2020). Dimethylaminomicheliolide is a novel chemotherapeutic agent that induces apoptosis and autophagy by adjusting the ROS/MAPK signaling pathway and inhibiting the Akt/mTOR signaling pathway to treat gliomas (Wang et al., 2019).

## Conclusion

Gliomas are highly malignant and prone to recurrence and progression. Although a certain degree of therapeutic effect can be achieved by applying standard treatment methods, the prognosis remains unsatisfactory. Oxidative stress has an important role in the occurrence and development of glioma, as well as in treatment. Therefore, antioxidative therapy can be considered a new therapeutic strategy for the treatment of gliomas. By summarizing the components of ROS, the role of oxidative stress in gliomas pathogenesis, the effects of oxidative stress on targets such as Nrf2, NOX2, NOX4, DGAT1, PKM2, PTPN2, PHB, OSMR, and PAX6 are presented in this paper, and some phytochemicals shown to alter glioma cell growth by affecting oxidative stress are discussed. Moreover, we suggest potential targets and drugs that modify radiosensitivity and chemoresistance by affecting oxidative stress, all of which provide new directions for our enriched treatment regimens for gliomas. However, extensive basic experimental and clinical trial research are still needed to explore the selection of intervention time and dosage of drugs. In addition, the efficacy of combining antioxidant treatment with other treatments also deserves to explore.

## Glossary

AgNTs: silver nanotriangles
AP-1: activator protein 1
APE1: apurinic/apyrimidinic endonuclease1
ARE: antioxidant response elements
ATP: adenosine triphosphate
Chk2: checkpoint kinase 2
CPT1A: carnitine palmitoyltransferase 1A
DGAT1: diacylglycerol-acyltransferase 1
DNA: deoxyribonucleic acid
ENPP2: ectonucleotide pyrophosphatase/phosphodiesterase 2
FAs: fatty acids
GBM: glioblastoma
GLUT1: glucose transporter 1
GSH: glutathione
GTP: guanosine triphosphate
HDAC: histone deacetylase
HGGs: high grade gliomas; 8-oxo-dG:8-oxo-7,8-dihydro-2′-deoxyguanosine
HIF-1: hypoxia-inducible factor-1
HIF-1α: hypoxia-inducible factor-1α
HMOX1: heme oxygenase 1
hMTH1: homolog protein 1
HO-1: heme oxygenase 1
IDH: isocitrate dehydrogenase
IFN: interferon
IL-1: interleukin-1; ATF4:Activating transcription factor 4
JAK2: janus kinase 2
KEAP1: kelch-like ECH-associated protein 1
LDHA: lactate dehydrogenase A
LGGs: low grade gliomas
MKK: MAPK kinase
MMPs: matrix metalloproteinases
NADH: nicotinamide adenine dinucleotide
NADPH: nicotinamide adenine dinucleotide phosphate
Nrf2: nuclear factor erythroid 2-related factor 2
OMV: outer-membrane vesicles
OSMR: oncostatin M receptor
PARP-1: poly ADP-ribose polymerase-1
PKM2: pyruvate kinase M2
PPVI: polyphyllin VI
PTPN2: protein tyrosine phosphatase, non-receptor type 2
Qu: quercetin
RBX1: RING box protein 1
RIP: receptor interacting protein kinase
ROS: reactive oxygen species
SAL: salinomycin
SeC: selenocysteine
SIRT6: sirtuin 6
SOD: superoxide dismutase
STAT3: signal transducer and activator of transcription 3
TGF: transforming growth factor
TNF: tumor necrosis factor
TQ: thymoquinone
TRAIL: TNF-related apoptosis-inducing ligand
VEGFA: vascular endothelial growth factor A
WHO: World Health Organization
YAP1: yes-associated protein 1
